# Apple Polyphenol Diet Extends Lifespan, Slows down Mitotic Rate and Reduces Morphometric Parameters in Drosophila Melanogaster: A Comparison between Three Different Apple Cultivars

**DOI:** 10.3390/antiox11112086

**Published:** 2022-10-22

**Authors:** Silvia Bongiorni, Ivan Arisi, Brunella Ceccantoni, Cristina Rossi, Camilla Cresta, Simona Castellani, Ivano Forgione, Sara Rinalducci, Rosario Muleo, Giorgio Prantera

**Affiliations:** 1 Department of Ecological and Biological Sciences (DEB), University of Tuscia, 01100 Viterbo, Italy; 2 Bioinformatics Facility European Brain Research Institute (EBRI) “Rita Levi-Montalcini”, and Institute of Translational Pharmacology National Research Council (CNR), Viale Regina Elena 295, 00161 Roma, Italy; 3 Department for Innovation in Biological, Agro-Food and Forest Systems (DIBAF), University of Tuscia, 01100 Viterbo, Italy; 4 Tree Physiology and Fruit Crop Biotechnology Laboratory, Department of Agriculture and Forest Sciences (DAFNE), University of Tuscia, 01100 Viterbo, Italy

**Keywords:** apple polyphenols, *Drosophila melanogaster*, model organisms, red flesh apple, lifespan, mitotic index, climbing assay

## Abstract

Plant-derived polyphenols exhibit beneficial effects on physiological and pathological processes, including cancer and neurodegenerative disorders, mainly because of their antioxidant activity. Apples are highly enriched in these compounds, mainly in their peel. The Tuscia Red (TR) apple variety exhibits the peculiar characteristic of depositing high quantities of polyphenols in the pulp, the edible part of the fruit. Since polyphenols, as any natural product, cannot be considered a panacea per se, in this paper, we propose to assess the biological effects of TR flesh extracts, in comparison with two commercial varieties, in a model system, the insect *Drosophila melanogaster*, largely recognized as a reliable system to test the in vivo effects of natural and synthetic compounds. We performed a comparative, qualitative and quantitative analysis of the polyphenol compositions of the three cultivars and found that TR flesh shows the highest content of polyphenols, and markedly, anthocyanins. Then, we focused on their effects on a panel of physiological, morphometrical, cellular and behavioral phenotypes in wild-type *D. melanogaster*. We found that all the apple polyphenol extracts showed dose-dependent effects on most of the phenotypes we considered. Remarkably, all the varieties induced a strong relenting of the cell division rate.

## 1. Introduction

Redox homeostasis represents a delicate balance between the production and removal of free radicals that plays critical roles in human health and is constantly regulated by oxidative stress and antioxidative defense systems [1] The overproduction of free radicals and reactive oxygen species (ROS), known as oxidative stress, can damage biomolecules, such as lipids, nucleic acids, proteins, causes cellular membrane peroxidation [2,3,4] and attracts various inflammatory mediators [5,6], leading directly or indirectly to severe diseases, such as cancer, atherosclerosis and neurological disorders [1,7,8]. Although the organisms possess endogenous systems to prevent radical-induced oxidative damage, they are sometimes insufficient to counteract extensive damage. Numerous epidemiological investigations evidenced the protection that increased consumption of fruit and vegetables exerts against cardiovascular diseases, various kinds of cancer, aging-related cognitive decline and other chronic diseases [9,10,11,12]. These properties were ascribed to the presence of phytochemicals, such as phenols [13]. Phenolic compounds are among the approximately 8000 secondary metabolites of plants that are synthesized in response to several adverse stimuli during plant development [14]. The beneficial effects of plant-derived polyphenols on a plethora of physiological and/or pathological processes, including cancer and neurodegenerative disorders, are well-known [15,16]. The main property of polyphenols is represented by the antioxidant activity they exert by scavenging free radicals normally produced by cell metabolism or in response to external factors [2,17,18,19]. Due to their richness in flavonoids and phenolic acids, apple fruits are considered one of the healthier fruits [20]. However, generally, apple polyphenols are enriched in the peel, and scarce and unstable in the pulp, which represents most of the edible part of the fruit. The red coloration of the apple fruit was ascribed to the accumulation of anthocyanins under the control of a MYB transcription factor [21,22,23].

The Tuscia Red apple variety (TR) was isolated in the experimental farm of the University of Tuscia by selection among a pool of seedlings generated from an open-pollinated old variety of Central Italy, having a red coloration of flesh and peel [24,25]. The origin of the old, red-fleshed variety could be ascribed to a lineage from the partially domesticated form of *M. sieversii* f. niedzwetzkyana, which contributed to give rise to the *M. domestica* var. niedzwetzkyana [23]. Apple species moved West along the ancient Silk Road, and the Romans had a preeminent role in the diffusion to Western Europe, from where they diversified and flourished worldwide [26]. TR combines the good taste of the domestic cultivar and the high polyphenol content of the pulp of *M. niedzwetzkyana*. 

In the present paper, we preliminary assessed the effects onto an in vivo model system of the polyphenol-rich TR apple pulp in comparison with the pulps of two commercial apple varieties, Annurca and Fuji. The fruit fly, *Drosophila melanogaster*, is a widely used model system in biomedical research [27,28,29] and an excellent model organism for the evaluation of drug toxicity and for the identification of pharmacological properties of plant-derived compounds [30,31,32].

The effects of the polyphenol pool extracted from the flesh of the three apple varieties were assessed on a group of biological parameters in *D. melanogaster*, including development timing, lifespan, starvation, morphometrical measures, cell division and locomotor abilities. 

## 2. Materials and Methods

### 2.1. Experimental Site and Plant Materials

The apple fruits were collected from an apple orchard located at the experimental farm of the Tuscia University in Viterbo, Italy. The apple orchard had Tuscia Red, Annurca and Fuji cultivars, grafted onto MM111 rootstock and grown as a spindle training system. The trees were planted in 2009, with intra- and inter-row distances of 3.0 m and 4.5 m, respectively. The harvesting time window was determined by visually inspecting the fruit exocarp color and maturation. The harvest was performed for each cultivar at apple commercial maturity, taking into consideration the assessment of fruit color, fruit firmness and starch index.

### 2.2. Apple Polyphenols Extraction

Polyphenols were extracted from three apple varieties, consisting of two commercial varieties, Annurca and Fuji, and one variety, the TR, obtained from open-pollination at the experimental agricultural farm at the Tuscia University. Three biological replicates were used for each extraction procedure. Three apples for each cultivar for each replicate were used.

Fresh fruit, deprived of peels, kept at 4 °C, were ground in a mortar under liquid nitrogen until a fine powder was obtained. The apple powder was immediately freeze-dried (EDWARDS modulo, Akribis Scientific Limited, Chester, UK), weighted and extracted in 5 mL of slightly acidified 70% methanol (pH = 4.00) for each gram of freeze-dried apple powder. The solution was sonicated for 10 s, vortexed and centrifuged at 15.000 rpm for 15 min. The pellet was separated from the supernatant, which was re-extracted in 5 mL per gram of slightly acidified 70% methanol (pH = 4.00), vortexed and centrifugated as above. The resulting supernatant was added to the supernatant previously obtained, filtered with 0.45 µm Whatman filters and evaporated by Rota-vapor at 35 °C under low pressure conditions. The remaining aqueous part of the solution was freeze-dried. Freeze-dried polyphenols were re-suspended in 3 mL of pure methanol for each gram of initial freeze-dried apple powder. The total phenolic content (TPC) of extracts was determined using the Folin–Ciocalteu (F–C) method. TPC was calculated from a calibration curve, using Gallic acids as a standard. Results are expressed as milligrams of Gallic acid equivalents (GAE) per 100 g of fresh weight of apple.

### 2.3. Total Anthocyanin Assay Method

Anthocyanins assay (AA) of the apple pulp was conducted according to Mancinelli et al. (1975) [33]. The grinded fresh sample (5 g), under liquid nitrogen, was placed in a test tube, and the anthocyanin fraction was extracted with 50 mL of 1% (*w*/*v*) HCl in methanol. The mixture was placed in the dark at 4 °C for 24 h, with continuous shaking. The extracts were cleared by filtration, and their absorbance at 530 nm was detected with a spectrophotometer and compared to a cyanidin-glucoside standard purchased by Extrasynthese (Genay, France).

### 2.4. HPLC Analysis

The total polyphenolic extract components, including phenolic acids (chlorogenic acid), dihydro-chalcones (phloretin-glucosides), anthocyanins (cyanidin-glucosides), flavonols (quercetin 3-O-ramnoside, quercetin 3-O-glucoside), flavanols (catechin and epicatechin) and procyanidins, were identified and quantified with reversed-phase liquid chromatography using a Dionex chromatograph (Dionex Corporation, Sunnyvale, CA, USA), equipped with a P680 quaternary pump, manual injector (Rheodyne) with 20 μL loop, TCC-100 thermostat oven and PDA-100 photodiode array detector, and controlled using Chromeleon software (version 6.50). The separation was carried out at 30 °C with a Dionex Acclaim^®^ 120 C18 column, 5 μm, 120 Å (4.6 × 250 mm). Optimized conditions were determined by flow injection analysis (FIA) of standard solutions at three different concentrations, ranging from 0 to 100 mg/L. Separations were performed by the mobile phase consisting of a ternary gradient: solvent A = 50 mM ammonium dihydrogen phosphate (99% purity) acidified to pH 2.6 with phosphoric acid (≥85% purity); solvent B = 20% solvent A and 80% acetonitrile (99.9% purity); solvent C = 0.2 M orthophosphoric acid (85% purity) acidified to pH 1.5 with NaOH (99.99% purity). All the HPLC reagents were supplied by the Sigma-Aldrich Chemical Co. (St. Louis, MO, USA). Samples were introduced into the column using a single injection with a 20 μL sample loop. The phenolic compounds were identified based on their elution order and the retention times of pure compounds and the characteristics of their UV–visible spectra. Catechin, epicatechin, procyanidin B1, procyanidin B2, quercetin-glucoside, cyanidin-glucoside, cyanidin 3-galactoside, phlorizin, chlorogenic acid, p-Coumaryc acid and caffeic acid standards were purchased at Extrasynthese (Genay, France).

### 2.5. Fruit Fly Strains and Treatments

All assays were performed using OrR wild-type stock obtained from the Bloomington Drosophila Stock Center (BDSC) at Indiana University (Bloomington, IN, USA). Flies were raised on a standard corn meal agar food (pH 5.5) at 25 °C. For the assays, which involved the exposure of embryos and larvae to apple polyphenols (APs), the fly diet was prepared by mixing the above standard food with 1.5, 3.0 or 5.0 mg/mL APs. For all the treatments, vials containing 10 mL of standard or AP-enriched diet were used. Experiments were conducted with only 1.5 and 3.0 mg/mL AP concentrations since it was preliminary assessed that the 5.0 AP concentration was harmful to the normal development and survival of flies.

One- to five-day-old flies were used in the experiments involving adult animals.

### 2.6. Developmental Assay

Larval development in all sets of treatments was checked every 24 h, and the number of pupae and emerging offspring, with dates of occurrence, were recorded.

### 2.7. Lifespan Assay

Flies were grown in either standard food or APs-enriched food from egg deposition to eclosure [34]. A total of 100 emerged offspring per treatment were collected and used for lifespan assays. Newly eclosed flies from each treatment were divided and housed in 10 vials (10 flies per vial). The flies were no longer exposed to APs-enriched food throughout their life. Every 2–3 days, flies were transferred to fresh food and deaths were recorded until all flies died. 

### 2.8. Stress Assay

A total of 100 flies per treatment were used for the starvation assay. Adult flies were housed in vials containing 2% agarose, to provide moisture, without any energy source. Deaths were recorded every 4 h. 

### 2.9. Size and Weight of Emerged Offspring

For size measurements, 100 flies per treatment were randomly selected from the population of emerged offspring. Fly length was measured from the top of the head to the end of the abdomen on graph paper. To determine weight, female and male adult individuals were separately weighed using a Sartorius SE2 Ultra Micro Balance (Bradford, MA, USA) in groups of 10 (n = 100 per treatment, per sex). 

### 2.10. Mitotic Index

Brains from third instar larvae were dissected in hypotonic solution (0.8% sodium citrate) and fixed at room temperature in a freshly prepared mixture of acetic acid/methanol/H2O (11:12:2) for 30 s. Single fixed brains were then transferred into small drops of 45% acetic acid on a clean, dust-free non-siliconized coverslip for 2 min. A clean slide was lowered onto the coverslip and gently squashed. The slide was frozen in liquid nitrogen, the coverslip was removed with a razor blade, and the slide was immediately immersed in absolute ethanol at −20 °C for 15 min. Slides were air-dried and stained with 0.2 μg/mL DAPI fluorochrome (Sigma-Aldrich Chemical Co, St. Louis, MO, USA) in 2x saline sodium citrate (20x = 0.15 M NaCl, 0.015 M sodium citrate) to detect chromatin and mitotic figures [35]. The mitotic index was defined as the number of mitotic cells per optical field [36]. The optical field was the circular area defined by a 100× Zeiss objective/1.30 Plan-NEOFLUAR, using 10× oculars and the Optovar set at 1.25 (Zeiss Axiophot microscope). Every optical field occupied by brain tissue was scored (three brains each slide, from at least 50–100 optical fields per slide per treatment). DAPI fluorescence was observed using 375/28× nm excitation filter (Chroma Technology Corp., Bellows Falls, VT, USA). 

### 2.11. Climbing Assay

The climbing ability test was conducted on young adult fruit flies. For each experiment, a group of 10 flies was placed in an empty vial and given 30 s to climb up. A horizontal line was drawn 17 cm above the bottom of the vial. The number of flies per treatment that could climb from the bottom to above the 17 cm mark in 10, 20 and 30 s was recorded as percentage of total flies. The test was performed three times in young adults between 1 and 5 days after eclosure. 

### 2.12. Statistical Analysis

Different statistical tests were applied to different experimental assays.

Total polyphenol content: Anova + post hoc LSD test; Anthocyanin content: Two-tailed Mann–Whitney U test; HPLC analysis: *t* test and two-tailed *t* test; Lifespan: Pairwise log-rank test, fdr correction; Starvation: Two-tailed Mann–Whitney U test, fdr correction; Larval length: Mann–Whitney U test, fdr correction; Larval weight: Two-tailed *t* test; Mitotic index: Fisher’s exact test, fdr correction; Climbing assay: Fisher’s exact test.

## 3. Results

The experiments reported in this study aimed to evaluate the effects of polyphenols extracted from three different apple varieties, TR, Annurca and Fuji, on several biological parameters. The lyophilized polyphenol extracts were diluted in the *Drosophila* standard food. The reported polyphenol concentrations refer to the final dilution of the extracts in 10 mL of standard corn meal agar food (see Material and Methods). In preliminary experiments, we assessed that the extract concentration of 5 mg/mL was harmful to the normal development and survival of flies, showing toxic effects that caused massive lethality. For this reason, we reported in detail only the results obtained with the concentrations of 1.5 and 3 mg/mL.

### 3.1. Apple Polyphenol Extracts Characterization

The scenario depicted by the total polyphenol content detected in the fruit flesh of the three apple cultivars (Table 1), highlighted that the TR flesh accumulates higher amounts of these compounds, more than 1.5 times that of the Annurca flesh content and three times that of Fuji flesh. In particular, the differences are even greater if we take into consideration the anthocyanin content detected in the fruit flesh of the three cultivars; therefore, the TR anthocyanin content (4 mg per 100 g of fresh weight) was 16 times greater than that of Annurca, whereas in Fuji, no anthocyanin content was detectable. The anthocyanin enrichment of TR fruit flesh is likely responsible of the red variegation of the pulp that can be clearly visualized in the longitudinal fruit sections of TR that is absent in the Fuji and Annurca pulp (Supplementary Figure S1).

Given the low total polyphenol content of Fuji flesh, further analytical analyses were limited to TR and Annurca apples. HPLC-DAD analysis results showed relevant differences in phenolic compound assortments of apple fruit flesh between TR and Annurca (Table 2). Hydroxy-cinnamic acids predominated in both varieties, principally due to the high levels of chlorogenic acid, whose concentration in TR fruit attains about double that of Annurca. Epicatechin and procyanidin B2 showed the highest concentrations in comparison to the other flavanols, while the catechin content was very low in both varieties. As a whole, the TR red fleshed fruits showed higher levels of total flavanols in comparison to Annurca (more than three folds). 

Flavonols represent only the smallest component (<1%) of the total polyphenolic content, with quercetin-glycosides the only flavonols found in the flesh of the two cultivars. 

As expected, phloretin-glycosides represented only a small part of the phenolic composition of the apple flesh in both varieties, but with interesting differences; phloretin-xyloglucoside showed much higher levels (5:1) in Annurca than in TR, while phlorizin (phloretin-glucoside) content in TR was double that of Annurca. 

Exclusive to TR was the accumulation of anthocyanin in fruit flesh. The main accumulated anthocyanins were cyanidin-3-galactoside, and to a minor extent cyanidin 3′-5′ diglucoside. 

### 3.2. Apple Polyphenols Do Not Impact Drosophila Development

The effect of apple polyphenol (APs) from Fuji, Annurca and TR apple on Drosophila development was evaluated by feeding embryos and larval stages with a normal diet (basal diet BD) supplemented with APs at two doses. We monitored the duration of the following stages: from egg laying until the emergence of the 3rd instar larvae, from the 3rd instar larvae to pupation, from pupation until the eclosion of adults. APs extracts from all cultivars at both stages produced no significant effects on larvae development, pupation and emergence of adults (data not shown).

### 3.3. Apple Polyphenols Extend Adult Fly Lifespan

**Figure 1:**
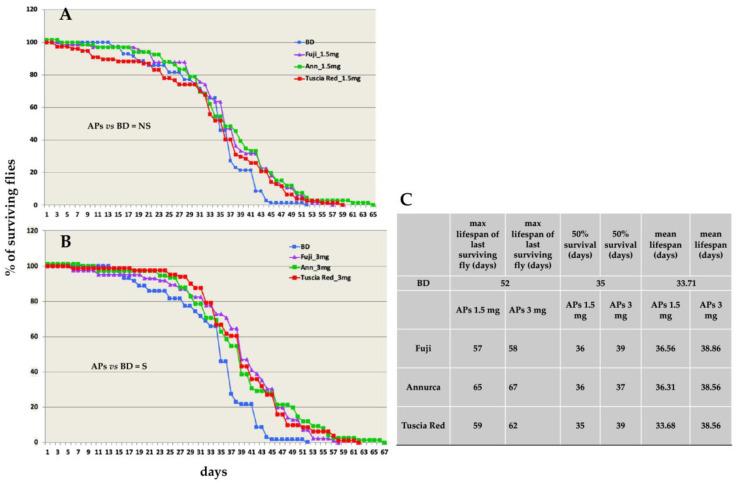
(**A**,**B**) The effects of 1.5 (**A**) and 3.0 (**B**) mg/mL APs from the three different apple cultivars on *D. melanogaster* lifespan. In the vertical axis, the number of surviving flies is reported; in the horizontal axis, days from the adult emergence until the fly death are reported. (**C**) In the table, the max lifespan of the last surviving individual, the day at which 50% of the original population still survived and the mean lifespan are reported. BD = basal diet with no supplement. NS = non-significant; S = significant. Statistical analysis (pairwise log-rank test, fdr correction) was applied to the differences in survival curves (3.0 mg/mL) between BD-fed and APs-fed flies. Tuscia Red vs. BD, *p* < 0.01; Fuji vs. BD, *p* < 0.001; Annurca vs. BD, *p* = 0.01.

To test the effect of apple APs on lifespan, flies were fed with APs from the three different cultivars at 1.5 and 3 mg/mL, during larval development only. The emerged offspring was transferred to normal food and the life duration was recorded as (i) day of the last surviving fly death, (ii) day of 50% survivors and (iii) mean lifespan. APs extracts from the three different cultivars at both concentrations showed a lifespan extension of the last surviving individual as compared to normally-fed flies (Figure 1), with the 3 mg/mL APs diet (Figure 1B) effect greater than that of 1.5 mg/mL diet (Figure 1A). 

However, statistical analysis showed that only the 3 mg/mL APs diet produced a significant increase in this parameter as compared to the basal diet (Figure 1C). 

### 3.4. Apple Polyphenols Ameliorate Fly Resistance to Caloric Restriction

The survival to food deprivation of 100 animals for each experimental point was recorded daily.

**Figure 2:**
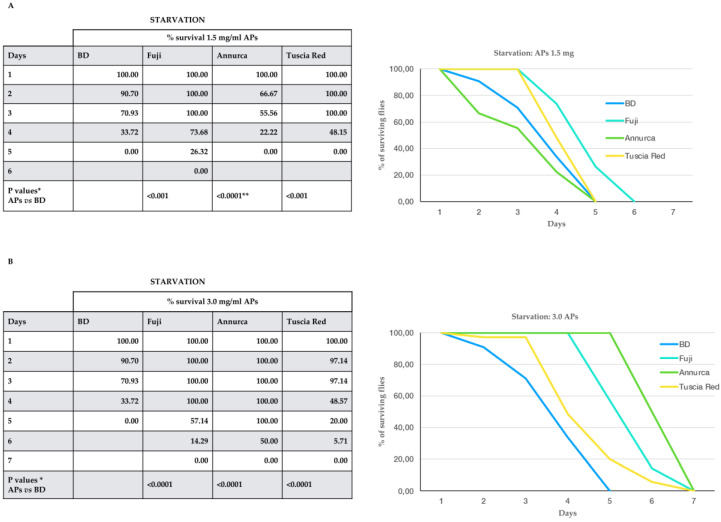
The effect of 1.5 (**A**) and 3.0 (**B**) mg/mL APs from the three different apple cultivars on *D. melanogaster* survival to starvation. Graphic representation of the data is on the right of each table. BD = basal diet with no supplement. All the differences between APs-supplemented diet vs. BD-fed animals were significant. * Mann–Whitney U test, fdr correction. ** Note that the Annurca extract *reduced* the resistance to starvation.

Flies that emerged from APs food at both 1.5 (Figure 2A) and 3 mg/mL (Figure 2B were significantly more resistant to starvation than normally-fed flies, except for 1.5 mg/mL Annurca polyphenol extracts, which significantly reduced the resistance to starvation as compared to normally-fed animals. Moreover, comparing the effects on starvation of the different apple varieties with each other showed significant differences (*p* < 0.001, pairwise comparisons) and a different ranking within the two concentrations, with Fuji > TR > Annurca with the lowest concentration and Annurca > Fuji > TR with the highest. 

### 3.5. Apple Polyphenol Effects on Offspring Size and Weight

To test the effects of APs from the three apple cultivars on *Drosophila* body morphological parameters, we measured the body length of larvae and the weight of adults. With regards to length, at least 60 larvae were measured (mm) for each experimental point. Third instar larvae grown on APs-supplemented food, showed a significant reduction in body length as compared to controls at both APs concentrations of the three apple cultivars (Table 3). 

Concerning weight, a significant reduction was observed in females fed with APs extracts from the three cultivars at both concentrations (*p* ≤ 0.01), except for TR polyphenol extracts at the lowest concentration (Table 4). Notably, no significant effect on the weight of adult males was observed with APs at either dose. As expected, weight scores of females were significantly higher than that of males (*p* < 0.001) in both BD and APs series.

### 3.6. Apple Polyphenols Affect Drosophila Mitotic Cell Cycle 

To analyze the mitotic cell cycle, brains from the 3rd instar larvae were dissected and fixed for subsequent cytological observation. The mitotic index was defined as the number of mitotic cells per optical field (see M&M). We observed a highly significantly (*p* < 0.001) reduced proliferation rate of neuroblasts in the larvae fed with both concentrations of APs, when compared to larvae fed with the basal diet (Table 5). The higher APs concentration showed a significantly more pronounced effect than the lower concentration of Annurca and TR APs (*p* < 0.05), but not of Fuji.

### 3.7. Apple Polyphenol Impact on Drosophila Climbing Ability

The climbing ability assay was conducted to evaluate the effect of APs extracts on locomotor function in fruit flies. The data showed that APs had only a slight effect on the motor skills of same-age adult flies, except for Annurca AP-fed flies, which showed a significantly reduced climbing ability, more pronounced with 3.0 mg/mL extracts (Table 6).

## 4. Discussion

The total polyphenol content in the TR apple variety was by far higher than that in Annurca and Fuji. This is particularly true for anthocyanin content (Table 1 and Table 2). As our analyses were performed on fruit flesh only, deprived of the peel, our observations clearly explain why the TR pulp is reddish (Figure S1) in comparison to the white pulp of the two other varieties.

Hydroxy-cinnamic acid predominance in the TR and Annurca varieties, as revealed by HPLC, is partially in contrast with other works [37,38], where the predominant class was flavanols. This difference could be partially explained by the slightly acidified extracting phase used, which could have major affinity to Hydroxy-cinnamics rather than to flavanols and also to the absence of peel in our fresh material for extracts. The accumulation of these phenolic acids is mainly responsible for the increased total phenolic content of TR and Annurca with respect to other white fleshed varieties. The predominance of flavanols among the flavonoid class in both TR and Annurca was in accordance with that found in other cultivars [37].

The main action of polyphenols in eukaryotic cells is that of scavenging reactive oxygen species (ROS), which may cause an imbalance in the redox homeostasis that finally results in oxidative stress [39].

The present results show that when apple polyphenols were added to the diet of laboratory populations of *D. melanogaster*, they affected a series of biological parameters, such as lifespan, resistance to starvation, morphometrical measures and mitotic index.

The APs-rich diet caused a slight but not significant delay in the sequential stages of animal development for a total of one day for each stage. In other words, the one-day delay accumulated by the 3rd instar larvae (the first monitored developmental stage) is maintained in later stages without any further retardation. The cause of this development delay is likely due to the slow-down of the cell cycle (see below).

Regarding the APs effects on the *Drosophila* lifespan (Figure 1), all the parameters we tested, i.e., mean life duration, the day at which 50% of flies still survived, and the day of death of the last surviving individual, were increased by the higher concentration (3.0 mg/mL) of polyphenols from all the apple cultivars we tested. The lower APs concentration (1.5 mg/mL) caused an extension of the lifespan of the last survivors but did not significantly affect the other two parameters. These results support those of Peng’s research group who demonstrated the effect of blueberry [40] and apple [41] polyphenols on the extension of the *Drosophila* lifespan.

Resistance to starvation and to oxidative stress are positively correlated [42]. Thus, it is not surprising that the AP-rich diet prolonged the fly survival to starvation (Figure 2). Intriguingly, Annurca extracts at the lowest concentration reduced the resistance to starvation with respect to normally-fed individuals, whereas at the highest ones, proved to be the most efficient AP to counteract starvation. Contrary to our results, Lopez et al. [34] reported that 10 mg/mL of green tea polyphenols sensitized flies to starvation. This discrepancy might be well-ascribed to the high dose of polyphenols used in Lopez’s experiments as compared to ours. In fact, we showed that our polyphenol extracts at the concentration of 5 mg/mL severely affected fly development and viability.

Morphometrical parameters were clearly affected by the APs-rich diet, which had an impact on both larval length and weight.

Flies fed with the APs-rich diet showed a reduction in the body length of the 3rd instar larvae, with respect to the normally-fed ones (Table 3). The difference is significant at both AP concentrations for all three cultivars. The two APs concentrations exhibited a clear dose–effect correlation (*p* < 0.001). The reduced larval size is fully consistent with the reduction in the body weight of adults, although with a significant difference between female and male individuals (Table 4).

In fact, both females and males showed a weight reduction with respect to basal diet-fed flies, but the difference was significant only for females with both concentrations, except for the lowest concentration of TR extracts. It is well-known that *D. melanogaster* male individuals have a body size consistently smaller than that of females and, hence, a body weight less than that of females [43]; thus, it makes sense that the APs-induced reduction in body weight is less pronounced in males than in females, since in the former the body weight cannot likely lower beyond a given survival threshold.

These results confirm and extend the observation of Lopez et al. [34], who reported a similar reduction in body size in flies fed with green tea polyphenol extracts, even though they used a higher concentration (10 mg/mL) than ours (1.5 and 3 mg/mL). Moreover, making these comparisons, it must be taken into account that the composition and content of polyphenols varies in green tea vs. apple extracts. By indirect evidence, the authors argue that the polyphenol-induced reduction in fly body size was due to a relenting of cell division rate and, hence, to the reduction in the body cell number [34]. Our results strongly reinforce this hypothesis that the decrease in cell division rate may be the cause of fly body size reduction since we actually observed a dramatic, statistically highly significant, drop-down of mitotic index in flies fed with the APs diet at both APs concentrations (Table 5). In this context, it must be emphasized that in *D. melanogaster,* the strict dependence of the diminution of body size from the decline in cell division rate is a well-known, common characteristics shared by *Minute* mutants [44], which harbor mutations in one of the genes encoding for ribosomal proteins [45,46]. The action of polyphenols on the mitotic cycle led many laboratories to investigate the effects of these compounds on cancer cells [47]. The inhibitory effect on the cell cycle progression was demonstrated in cancer cells in culture for green and black tea and rosemary polyphenols [48,49,50]. Many studies demonstrated that polyphenols derived by different plants may arrest cell cycle progression in metaphase or cause mitotic catastrophe [51,52,53]. Our observations contrast with the above results, since polyphenols from all three apple cultivars induced a relenting not an arrest of the cell cycle, as shown by a dramatic reduction in the number of mitotic figures instead of an accumulation of metaphases. Additionally, the few mitotic figures we observed were appeared normal, with no evidence of mitotic catastrophe. Moreover, as far as we know, the demonstration of a similar effect of polyphenols on the cell cycle of normal cells in vivo was never reported in the literature.

As a behavioral test of the APs diet effect, we took into consideration the climbing ability of *D. melanogaster* adults (Table 6). Only Annurca APs negatively impacted the climbing ability. The literature data on the effects of polyphenols on climbing ability were mostly focused on the rescue of pathological, locomotory phenotypes of neurodegenerative disorders that were mimicked in the fruit flies either by chemical treatment [40,41] or by transgenic manipulation [54]. Studies on wild-type *D. melanogaster* adults demonstrated that the apple-derived polyphenol Phlorizin is able to partially rescue the age-related climbing ability decline [55]. However, the effect of phlorizin did not appear before 30 days of age of the animals; thus, it is not surprising that the locomotor skills of the young animals (1–5 days old) used in our experiments were not influenced by APs extract feeding.

A comparative analysis of the effects of the three cultivars (see synoptic Table 7), evidenced a general uniformity of effects on the considered characters, the most striking difference being the just mentioned negative effect exerted by Annurca polyphenol extracts on locomotory skills. This adverse effect could be ascribed to some cultivar-specific metabolite, which can be resolved by a high throughput, differential metabolomic analysis (manuscript in preparation).

In conclusion, looking for a possible primary effect underlying the other effects exerted by apple polyphenols, it can be considered that the drastic reduction in mitotic index may well be responsible for the diminution in both body size and adult weight. The correlation between mitotic index and lifespan is more obscure. However, evidence was reported in yeast that the cell division rate is inversely related to the lifespan of the individual [56,57]. Even though considering that yeast is a unicellular eukaryote, the above observations support our ones in that APs induced both a dramatic slowing of the cell cycle and a significant increase in the lifespan.

## Figures and Tables

**Table 1:** Total polyphenol and anthocyanin detected in the flesh of apple fruit of the cultivars studied. The content of polyphenol is expressed as mg of Gallic Acid equivalent for 100 g of fresh weight of flesh. The content of anthocyanin is expressed as mg equivalent of cyanidin 3-glucoside for 100 g of fresh weight of flesh. Values represent the average ± S.D. For total polyphenol content, Anova + post hoc LSD test was used to assess the significance of the observed differences in the three cultivars. Anova: F = 89.621, *p* < 0.001; a vs. b, a vs. c, and b vs. c: LSD *p* ≤ 0.001; For the difference in Anthocyanin content: d vs. e *p* < 0.001, Mann–Whitney U test.

Compound	Fuji	Tuscia Red	Annurca
Total Polyphenol (mg GAE/100 g fw)	101.7 ± 6.8 a	316.8 ± 28.4 b	183.6 ± 39.7 c
Anthocyanin content (mg Cy3Glu/100 g fw)	N.D.	4.234 ± 0.643 d	0.256 ± 0.078 e

**Table 2:** Polyphenolic content and identification by HPLC analysis in Annurca and Tuscia Red fruit flesh. Numbers following the ± symbol indicate the standard deviation. Values are expressed as mg of compound per 1 kg of fresh weight. Values significantly different (*p* < 0.05) between Tuscia Red and Annurca are indicated by * (two-tailed *t* test) or by ** (*t* test), which are placed to the side of the higher value. Examples of chromatograms of three representative compounds in the two cultivar fleshes are reported in Supplemental Figure S2.

Compounds (mg/Kg fw)	Apple Variety	
	Annurca	Tuscia Red
Cholorogenic acid	1439.2 ± 225.7	2427.5 ± 574.2
Coumaryl-quinic acid	203.4 ± 69.6	491.2 ± 160.9 *
Other hydroxyl-cinnamics	39.4 ± 15.4	68.4 ± 28.8
Total hydroxyl-cinnamics	1682.1± 310.7	2987.1 ± 763.9
Catechin	3.7 ± 1.2	17.2 ± 7.3 *
Epicatechin	107.5 ± 24.6	317.8 ± 110.2 *
Procyanidin B1	71.4 ± 21.4	194.7± 56.8 *
Procyanidin B2	146.5 ± 30.7	498.8 ± 169.0 *
Other procyanidins	81.4 ± 2.2	465.8 ± 33.7 *
Toal flavanols and procyanidins	410.5 ± 80.2	1494.3 ± 377.0 *
Cyanidin 3′-5′ diglucoside	0.0	12.6 ± 6.4 **
Cyanidin 3′-galactoside	0.0	64.1 ± 27.6 **
Other cyanidins	0.0	6.6 ± 0.3 **
Total anthocyanin	0.0	83.3 ± 34.2 **
Quercitin 3′ glucoside	2.8 ± 2.3	11.5 ± 6.6
Quercitin 3′ ramnoside	5.5 ± 4.0	11.6 ± 6.9
Total flavonols	8.4 ± 6.4	23.1 ± 13. 0
3′ hydroxyl-phloretin-glucoside	52.5 ± 10.2	42.2 ± 11.8
3′ phloretin-xyloglucoside	119.5 ± 42.4 *	23.5 ± 12.8
Phlorizin	31.4 ± 16.9	62.1 ± 27.0
Total di-hydro chalcones	203.4 ± 69.5	127.8 ± 41.7
Total polyphenols	2326.2 ± 466.7	4617.9 ± 1166.3 *

**Table 3:** The effect of 1.5 mg/mL (A) and 3.0 mg/mL (B) APs from the three different apple cultivars on *D*. *melanogaster* 3rd instar larvae length. BD = basal diet with no supplement. * Mann–Whitney U test, fdr correction: APs *vs* BD.

A				
	BD	Fuji	Tuscia Red	Annurca
Larval length	3.9	3.7	3.8	2.9
*p* values * APs vs. BD		<<0.001	<0.001	<<0.001
**B**				
	**BD**	**Fuji**	**Tuscia Red**	**Annurca**
Larval length	3.9	3.0	2.7	2.6
*p* values * APs *vs* BD		<<0.001	<<0.001	<<0.001

**Table 4:** The effect of 1.5 (A) and 3.0 (B) mg/mL APs from the three different apple cultivars on *D*. *melanogaster* adult weight. BD = basal diet with no supplement. * The weight of groups of ten male or female individuals is reported. At least 8 groups for each experimental point were recorded. ^s^ and ^ns^ denote whether or not the mean weight difference between the BD-fed and APs-fed flies is significant (*p* ≤ 0.01, two-tailed *t* test). Male mean weight is significantly lower than that of females (*p* < 0.001, two-tailed *t* test).

A								
	Weight (g) *							
	BD		Fuji		Tuscia Red		Annurca	
	females	males	females	males	females	males	females	males
Average	0.0085	0.0055	0.0070 ^s^	0.0048 ^ns^	0.0079 ^ns^	0.0055 ^ns^	0.0062 ^s^	0.0052 ^ns^
SD	0.0010	0.0014	0.0014	0.0008	0.0018	0.0009	0.0009	0.0015
**B**								
	Weight (g) *							
	**BD**		**Fuji**		**Tuscia Red**		**Annurca**	
	females	males	females	males	females	males	females	males
Average	0.0085	0.0055	0.0069 ^s^	0.0053 ^ns^	0.0071 ^s^	0.0052 ^ns^	0.0069 ^s^	0.0049 ^ns^
SD	0.0010	0.0014	0.0014	0.0016	0.0005	0.0014	0.0010	0.0004

**Table 5:** The effect of 1.5 (A) and 3.0 (B) mg/mL APs from the three different apple cultivars on *D*. *melanogaster larval* brain mitotic index. BD—basal diet with no supplement. *P*—Fisher’s exact test, fdr correction.

A				
APs 1.5 mg	Mitotic Divisions	Optical Fields	Mitotic Index	*p* APs vs. BD
BD	216	200	1.08	
Fuji	51	220	0.23	<<0.001
Annurca	79	308	0.26	<<0.001
Tuscia Red	88	306	0.29	<<0.001
**B**				
**APs 3 mg**	**Mitotic Divisions**	**Optical Fields**	**Mitotic Index**	***p*** **APs vs. BD**
BD	216	200	1.08	
Fuji	56	290	0.19	<<0.001
Annurca	31	200	0.15	<<0.001
Tuscia Red	37	200	0.18	<<0.001

**Table 6:** The effect of 1.5 (A) and 3.0 (B) mg/mL APs from the three different apple cultivars on adult *D. melanogaster* climbing ability. T is indicated as the time in seconds from the start of the experiment when all the flies were at the bottom of a graduated cylinder. The % values refer to the percentages of flies achieving the height of 17.5 cm in the indicated time. The test was reproduced three times with flies of the same age. BD—basal diet with no supplement. *p* value APs vs. BD, Fisher’s exact test: (*) *p* < 0.05, (**) *p* < 0.01, (***) *p* < 0.001.

A			
APs 1.5 mg	T10%	T20%	T30%
BD	85	90	100
Fuji	80	95	100
Annurca	40 (*)	95	100
Tuscia Red	90	90	100
**B**			
**APs 3.0 mg**	**T10%**	**T20%**	**T30%**
BD	85	90	100
Fuji	35 (**)	90	100
Annurca	5 (***)	50 (*)	65 (*)
Tuscia Red	70	100	100

**Table 7:** Synopsis of the effects of the three different apple varieties at two concentrations (1.5 and 3.0 mg/mL) on various *D. melanogaster* biological parameters. Data were compared to those of basal diet-fed animals and only significant values were considered. + indicates an incremental by APs diet effect on the considered parameter; – indicates a lowering and – – a highly lowering effect on the considered parameter; 0 indicates no effect on the considered parameter.

	Annurca		Fuji		Tuscia Red	
	1.5	3.0	1.5	3.0	1.5	3.0
Development timing	0	0	0	0	0	0
Mean lifespan	0	+	0	+	0	+
Resistance to starvation	–	+	+	+	+	+
Larval size	–	–	–	–	–	–
Weight (females)	–	–	–	–	0	–
Mitotic index	– –	– –	– –	– –	– –	– –
Climbing ability	–	–	0	0	0	0

## Data Availability

Data are contained within the article and supplementary material.

## Supplementary Materials

The following supporting information can be downloaded at: https://www.mdpi.com/article/10.3390/antiox11112086/s1, Figure S1: Comparison of the fruits of the three apple varieties used in the study; Figure S2: Examples of chromatograms for three representative compounds extracted from the pulp of Tuscia Red and Annurca apple varieties.
