# Supplementary material for: Apple Polyphenol Diet Extends Lifespan, Slows down Mitotic Rate and Reduces Morphometric Parameters in Drosophila Melanogaster: A Comparison between Three Different Apple Cultivars
Source: Antioxidants (Basel). 2022 Oct 22;11(11):2086. doi: 10.3390/antiox11112086 (PMC9686679; doi:10.3390/antiox11112086)
Supplement: Supplementary file 1 [file antioxidants-11-02086-s001.zip › Supplemental Figure S1.pptx]

## Slide 1
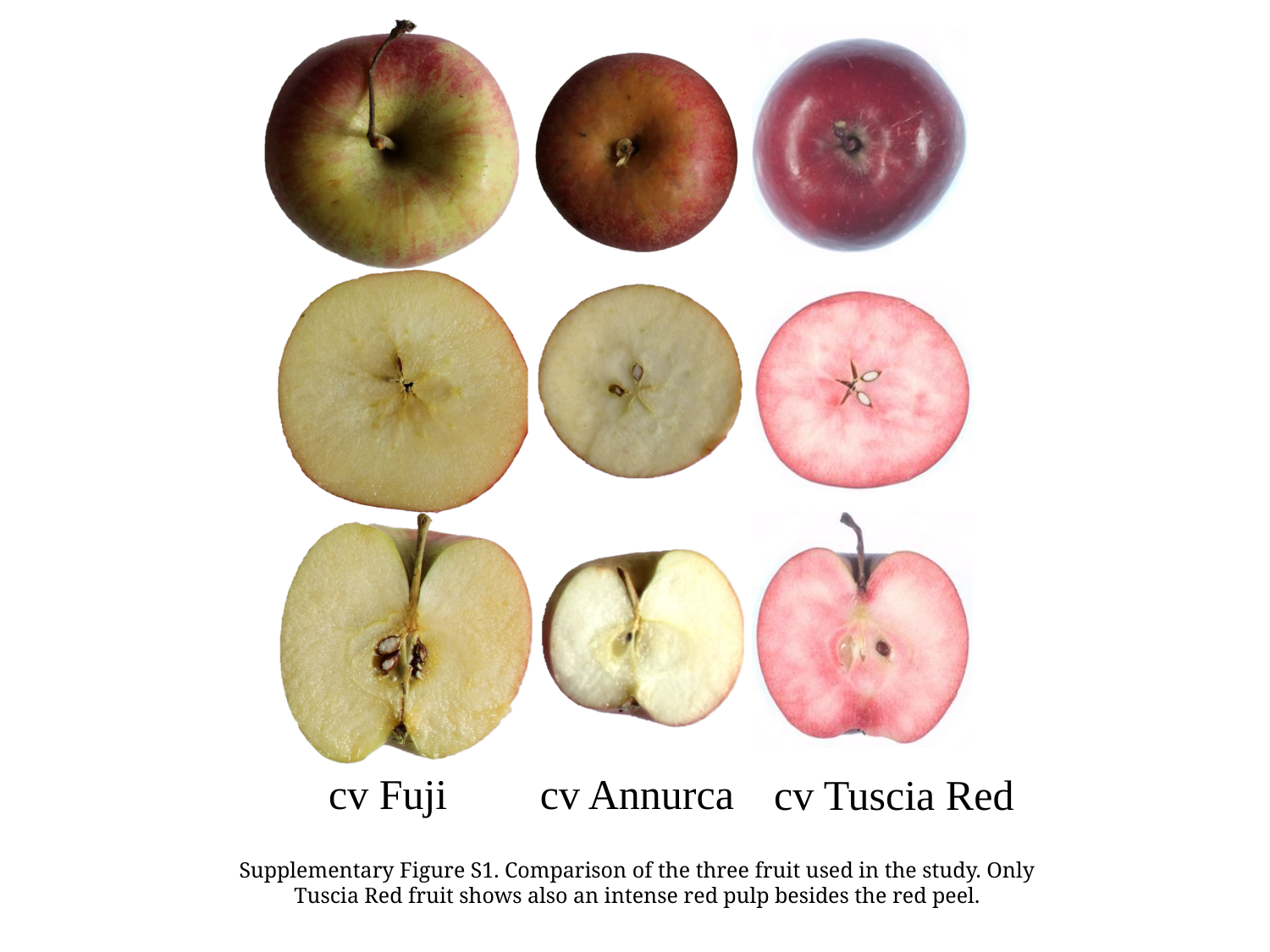

cv Annurca
cv Fuji
cv Tuscia Red
Supplementary Figure S1. Comparison of the three fruit used in the study. Only Tuscia Red fruit shows also an intense red pulp besides the red peel.
