# Supplementary material for: Apple Polyphenol Diet Extends Lifespan, Slows down Mitotic Rate and Reduces Morphometric Parameters in Drosophila Melanogaster: A Comparison between Three Different Apple Cultivars
Source: Antioxidants (Basel). 2022 Oct 22;11(11):2086. doi: 10.3390/antiox11112086 (PMC9686679; doi:10.3390/antiox11112086)
Supplement: Supplementary file 1 [file antioxidants-11-02086-s001.zip › Supplemental Figure S2.pptx]

## Slide 1
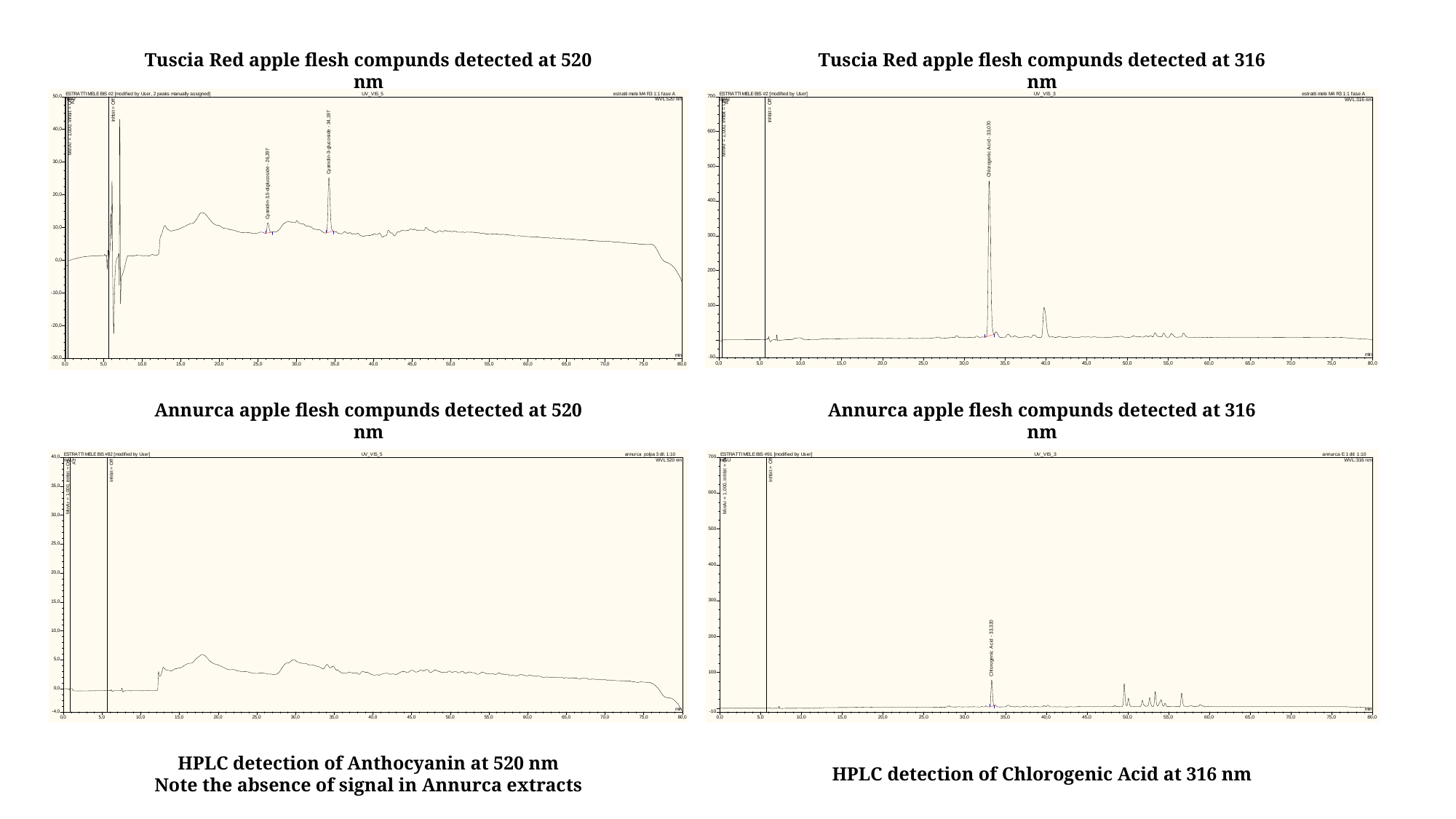

Tuscia Red apple flesh compunds detected at 316 nm
Tuscia Red apple flesh compunds detected at 520 nm
Annurca apple flesh compunds detected at 520 nm
Annurca apple flesh compunds detected at 316 nm
HPLC detection of Anthocyanin at 520 nm
Note the absence of signal in Annurca extracts
HPLC detection of Chlorogenic Acid at 316 nm
